# Triple Negative Breast Tumors in African-American and Hispanic/Latina Women Are High in CD44+, Low in CD24+, and Have Loss of PTEN

**DOI:** 10.1371/journal.pone.0078259

**Published:** 2013-10-22

**Authors:** Yanyuan Wu, Marianna Sarkissyan, Yahya Elshimali, Jaydutt V. Vadgama

**Affiliations:** 1 Division of Cancer Research and Training, David Geffen UCLA School of Medicine, Los Angeles, California, United States of America; 2 Department of Internal Medicine, David Geffen UCLA School of Medicine, Los Angeles, California, United States of America; 3 Charles R. Drew University of Medicine and Science, David Geffen UCLA School of Medicine, Los Angeles, California, United States of America; 4 Jonsson Comprehensive Cancer Center and David Geffen UCLA School of Medicine, Los Angeles, California, United States of America; The University of Arizona, United States of America

## Abstract

**Background:**

African-American women have higher mortality from breast cancer than other ethnic groups. The association between poor survival and differences with tumor phenotypes is not well understood. The purpose of this study is to assess the clinical significance of (1) Stem cell-like markers CD44 and CD24; (2) PI3K/Akt pathway associated targets PTEN, activation of Akt, and FOXO1; and (3) the Insulin-like growth factor-1 (IGF-I) and IGF binding protein-3 (IGFBP3) in different breast cancer subtypes, and compare the differences between African-American and Hispanic/Latina women who have similar social-economic-status.

**Methods:**

A total of N=318 African-American and Hispanic/Latina women, with clinically-annotated information within the inclusion criteria were included. Formalin fixed paraffin embedded tissues from these patients were tested for the different markers using immunohistochemistry techniques. Kaplan-Meier survival-curves and Cox-regression analyses were used to assess Relative Risk and Disease-Free-Survival (DFS).

**Results:**

The triple-negative-breast-cancer (TNBC) receptor-subtype was more prevalent among premenopausal women, and the Hormonal Receptor (HR) positive subtype was most common overall. TNBC tumors were more likely to have loss of PTEN, express high Ki67, and have increased CD44+/CD24- expression. TNBC was also associated with higher plasma-IGF-I levels. HR-/HER2+ tumors showed high pAkt, decreased FOXO1, and high CD24+ expression. The loss of PTEN impacted DFS significantly in African Americans, but not in Hispanics/Latinas after adjusted for treatment and other tumor pathological factors. The CD44+/CD24- and CD24+/CD44- phenotypes decreased DFS, but were not independent predictors for DFS. HER2-positive and TNBC type of cancers continued to exhibit significant decrease in DFS after adjusting for the selected biomarkers and treatment.

**Conclusions:**

TNBC incidence is high among African-American and Hispanic/Latino women residing in South Los Angeles. Our study also shows for the first time that TNBC was significantly associated with PTEN loss, high Ki67 and the CD44+/CD24- phenotype. The loss of PTEN impacts DFS significantly in African Americans.

## Introduction

African-American and Hispanic/Latina women with breast cancer have poor outcome compared with other groups [1]. Studies have suggested that differences in tumor receptor-subtypes may potentially play a role in the disparate outcomes from breast cancer observed among African-American and Hispanic/Latina women [2,3]. Since the expression of conventional receptor markers ER, PR, HER2 and the proliferative marker, Ki67, guide treatment regimes, they may also play a significant role in predicting outcome [4]. Notably, women with TNBC have the most limited treatment options since targeted receptor therapies such as Tamoxifen for HR+ tumors, and Trastuzumab for HER2+ tumors would not be recommended [5] . Further compounding the health disparity issue is that the TNBC tumor subtype is more prevalent in African-American women, especially premenopausal, compared to European-American/Caucasian women [2,6-8]. Hence, clinical evaluation of tumor receptor subtypes and biomarkers in relation to outcome among underrepresented populations is strongly warranted particularly since there is a dearth of data available compared to information available among European-American/Caucasian women. 

Recent studies investigating gene and protein profiles of tumors from large cohorts have revealed several candidate profiles that may be associated with tumor subtypes and outcomes [9]. Studies from our group, and others, have identified that the pathway directed along the Insulin-like Growth Factor (IGF) axis (specifically involving IGF-I, IGFBP3, down the PI3K/Akt cascade to FOXO1) is strongly associated with breast cancer outcomes. The IGF pathway is well-characterized in tumorogenesis. IGF-I acts as a systemic growth factor and is bound by several binding proteins, particularly Insulin-like Growth Factor Binding Protein (IGFBP)-3 which sequesters and limits its bioavailability. Our laboratory and others have demonstrated a significant association of high IGF-I circulatory levels, low IGFBP-3 levels, and a correlation with breast cancer risk and outcome [10-14], notably among African-American and Hispanic/Latina women [11]. 

The central mechanism whereby IGF-I contributes to breast cancer formation and progression is mediated through the IGF-I Receptor (IGF-IR) on the breast cell membrane which subsequently activates signal transduction via the MAPK and PI3K/Akt pathways [15]. The PI3K/Akt pathway plays a key role in breast cell growth, proliferation, survival and metabolism [16]. Aberrations in PI3K/Akt signaling such as loss of PTEN, an Akt phosphorylation inhibitor and tumor suppressor, as well as overexpression and over-activation of Akt (pAkt), have been identified as important mechanisms contributing to breast cell proliferation [16-19]. Our previous studies had identified increased activation of Akt and poor clinical outcome among African-American and Hispanic women with breast cancer [17]. In addition, studies from our group also reported that loss of FOXO1, a downstream protein within the Akt signaling pathway, is associated with reduced breast cancer outcome among underrepresented women [20]. Therefore, investigation of several biomarkers in the AKT/PI3K pathway, including PTEN, pAkt, and FOXO1 as a part of a biomarker panel has strong clinical and molecular basis. We added IGF-I and IGFBP-3 circulatory levels as biomarkers for analysis since it has been confirmed as a clinically relevant prognostic biomarker and is an upstream activator of the PI3K/Akt pathway [15].

**Table 1:** Prevalence of Breast Cancer Receptor-Subtypes by Ethnicity, Age, and Tumor Histopathology.

**CHARACTERISTICS**			**RECEPTOR-SUBTYPE**					**P-VALUE**	
		**All Cases**	**HR+/HER2-**	**HR+/HER2+**	**HR-/HER2+**	**TNBC**			
		**N (%)**	**N (%)**	**N (%)**	**N (%)**	**N (%)**			
		**318 (100)**	**156 (49.1)**	**29 (9.0)**	**32 (10.1)**	**101 (31.8)**			
**Ethnicity**								
	African-American	166 (52.2)	82 (49.4)	15 (9.0)	11 (6.6)	58 (35.0)		
	Hispanic/Latina	152 (47.8)	74 (48.7)	14 (9.2)	21 (13.8)	43 (28.3)		0.721
**Age Characteristics**								
Age, mean yrs (SD)		50.6 (10.2)	51.7 (10.3)	51.5 (10.0)	50.0 (10.1)	48.8 (10.1)		0.159
Age groups								
	< 50 yrs	153 (48.1)	65 (42.5)	14 (9.2)	15 (9.8)	59 (38.5)		
	> 50 yrs	165 (51.9)	91 (55.2)	15 (9.1)	17 (10.3)	42 (25.4)		0.011*
African-American								
	< 50 yrs	68 (41.0)	30 (44.1)	7 (10.3)	2 (3.0)	29 (42.6)		
	> 50 yrs	98 (59.0)	52 (53.1)	8 (8.2)	9 (9.2)	29 (29.5)		0.185
Hispanic/Latina								
	< 50 yrs	85 (55.9)	35 (41.2	7 (8.2)	13 (15.3)	30 (35.3)		
	> 50 yrs	67 (44.1)	39 (58.2)	7 (10.4)	8 (12.0)	13 (19.4)		0.015*
**Tumor Histopathology**								
**AJCC stage (N = 303)**								
	0-I	69 (22.8)	40 (26.3)	9 (31.03)	7 (22.6)	13 (14.3)		
	II	139 (45.9)	67 (44.1)	7 (24.14)	14 (45.2)	51 (56.0)		0.220
	III-IV	95 (31.3)	45 (29.6)	13 (44.83)	10 (32.2)	27 (29.7)		
**Tumor size (N = 303)**								
	<2cm	95 (31.4)	54 (35.3)	10 (34.5)	10 (33.3)	21 (23.0)		
	2cm - 5cm	126 (41.6)	68 (44.4)	7 (24.1)	9 (30.0)	42 (46.2)		0.022*
	>5cm	82 (27.0)	31 (20.3)	12 (41.4)	11 (36.7)	28 (30.8)		
**Lymph node (N = 305)**								
	Positive	165 (54.1)	78 (51.0)	16 (55.2)	15 (50.0)	56 (60.2)		0.199
	Negative	140 (45.9)	75 (49.0)	13 (44.8)	15 (50.0)	37 (39.8)		
**Histology (N=303)**								
	IDC/ILC	244 (80.53)	123 (81.5)	26 (89.7)	22 (73.3)	73 (78.5)		
	DCIS/LCIS	48 (15.84)	24 (15.9)	2 (6.9)	6 (20.0)	16 (17.2)		0.345
	Inflammatory/other	11 ( 3.63)	4 ( 2.6)	1 (3.4)	2 ( 6.7)	4 (4.3)		
**Differentiation (N=298)**								
	Well	34 (11.4)	21 (14.1)	6 (20.7)	2 ( 6.7)	5 (5.6)		
	Moderately	96 (32.2)	61 (40.9)	7 (24.1)	13 (43.3)	15 (16.7)		<0.001*
	Poorly	168 (56.4)	67 (45.0)	16 (55.2)	15 (50.0)	70 (77.7)		
**Ki67 (N=213)**								
	Low	137 (64.3)	84 (80.8)	15 (62.5)	15 (68.2)	23 (36.5)		<0.001*
	High	76 (35.7)	20 (19.2)	9 (37.5)	7 (31.8)	40 (63.5)		

Abbreviations: HR – Hormonal Receptor (Estrogen/Progesterone Receptors), HER2 ( human epidermal growth factor receptor 2), TNBC – Triple Receptor-Negative Breast Cancer (ER-/PR-/HER2-) , SD – Standard Deviation, AJCC –American Joint Committee on Cancer, IDC/ILC – Infiltrating Ductal/Lobular Carcinoma; DCIS/LCIS – Ductal/Lobular Carcinoma In Situ. * P-value < 0.05 is significant.

A third central component which needs to be taken into consideration when assessing biomarkers for clinical outcome is drug-therapy resistance. The presence of cancer-stem-cell-like cells (CSC’s) has been implicated significantly in therapy resistance in breast cancer. The breast CSC phenotype is most commonly described as expression of the markers: CD44 positive (CD44+) and CD24 low/negative (CD24-). As biomarkers, CD44+/CD24-/low breast cells have been shown to have tumor-initiating properties in breast cancer [21], show enhanced invasive properties [22], and are more likely to become resistant to radiation therapy [23]. Clinically, tumors with a higher fraction of CD44+/CD24- cells were more commonly found in patients diagnosed with distant metastases [24] and have been associated with poor clinical outcome [25]. Recent studies have identified that TNBC breast tumors may also have a higher content of CD44+/CD24- cells [22,24,26,27]. Unfortunately, there is a paucity of data regarding the prevalence of the CD44/CD24 phenotype as a biomarker in breast tumor tissues and in relation to tumor clinicopathology among African-American and Hispanic women with breast cancer. Therefore, CD44 and CD24 are important representative CSC biomarkers which have been added to the panel. 

Examination of predictive biomarkers representative of several key pathways implicated in breast cancer outcome will elucidate understanding of the development of breast tumors in the context of receptor subtypes. The majority of studies to date have been conducted on European-American/Caucasian women, hence investigations focused on African-American and Hispanic women with breast cancer is particularly important due to high degree of disparity in these groups. The key objectives of our current study were: (a) characterize differences in tumor subtypes among African-American and Hispanic/Latina patients; (b) identify potential biomarkers associated with tumor progression within each tumor subtype (especially, in TNBC); and (c) determine outcome in breast cancer patients with different biomarker expression and tumor subtypes. 

## Methods

### Patients

Patients were selected from an ongoing breast cancer study conducted in the Division of Cancer Research at Charles R. Drew University. Women were informed and consented from Martin Luther King Ambulatory Care Center (MACC) between 1995 and 2007. This study was approved by the Charles R. Drew University of Science and Medicine Institutional Review Board and written informed consent was obtained from all participants (Approval 00-06-041-13). The consent protocol was reviewed and approved by our Institutional Review Board. A total 1400 participants have been consented into the study, and 370 subjects have breast cancer confirmed by surgical biopsy/pathology and follow-up data. Among the 370 subjects, 94% (N=347) self-identified as African-American and Hispanic/Latina. The majority of subjects, ~84% of African-American and ~91% Hispanic/Latina, reported having no health insurance. An additional 6% of the participants self-identified as either Caucasian (3%, N = 12) or Asian (3%, N = 11) and these women were omitted from the study due to small numbers. Among the N=347 participants, N=29 women had no breast tumor receptor status available and were also omitted. Therefore, the final number of participants included in the analysis of the study was N=318, with 52.2% self-identified as African-American and 47.8% as Hispanic/Latina. 

**Table 2:** Biomarker Expression in Tissue and Plasma in Relation to Breast Cancer Receptor Subtypes.

**CHARACTERISTICS**			**RECEPTOR-SUBTYPE**					**P-VALUE**
			**HR+/HER2-**	**HR+/HER2+**	**HR-/HER2+**	**TNBC**		
			**N (%)**	**N (%)**	**N (%)**	**N (%)**		
**CD44**								
	Positive (n=60)		24 (40.0)	9 (15.0)	5 ( 8.3)	22 (36.7)		0.025*
	Negative (n=66)		36 (54.4)	10 (15.2)	10 (15.2)	10 (15.2)		
**CD24**								
	Positive (n=75)		30 (40.0)	14 (18.7)	12 (16.0)	19 (25.3)		0.213
	Negative (n=51)		30 (58.8)	5 ( 9.8)	3 ( 5.9)	13 (25.5)		
**CD44+/CD24-**								
	Positive (n=37)		10 (27.0)	4 (10.8)	3 ( 8.1)	20 (54.1)		<0.001*
	Negative (n=89)		50 (56.2)	15 (16.8)	12 (13.5)	12 (13.5)		
**CD24+/CD44-**								
	Positive (n=50)		18 (36.0)	9 (18.0)	10 (20.0)	13 (26.0)		0.132
	Negative (n=76)		42 (55.3)	10 (13.1)	5 (6.6)	19 (25.0)		
**PTEN**								
	Positive (n=80)		46 (57.5)	8 (10.0)	11 (13.8)	15 (18.7)		0.002*
	Negative (n=62)		17 (27.4)	14 (22.6)	8 (12.9)	23 (37.1)		
**pAkt**								
	Negative (n=62)		33 (53.2)	9 (14.5)	7 (11.3)	13 (21.0)		0.056
	Positive (n=64)		21 (32.8)	13 (20.3)	12 (18.8)	18 (28.1)		
**FOXO1**								
	Positive (n=59)		34 (57.6)	9 (15.3)	6 (10.2)	10 (16.9)		0.002*
	Negative (n=80)		28 (35.0)	11 (13.8)	10 (12.5)	31 (38.7)		

**CHARACTERISTICS**			**MEAN±SD**	**MEAN±SD**	**MEAN±SD**	**MEAN±SD**		**TOTAL**

**Plasma IGF-I** (ng/ml)								
	Premenopausal		118±71	112±61	92.5±55	134±68		137±69
	Postmenopasual		112±52	117±68	110±48	122±68		116±65
			**P-Value=0.053**	P-Value=0.902	P-Value=0.994	P-Value=0.318		P-Value=0.0318*
**Plasma IGFBP3** (μg/ml)								
	Premenopausal		2.8±1.2	3.1±0.9	2.8±1.7	2.9±0.9		2.9±0.9
	Postmenopasual		3.0±0.9	2.7±1.5	3.8±0.5	2.9±1.0		2.8±1.1
			P-value=0.432	P-value=0.696	P-value=0.223	P-value=0.850		P-value=0.850

Abbreviations: HR–Hormonal Receptor (Estrogen/Progesterone Receptor), HER2 (human epidermal growth-factor-receptor 2), TNBC – Triple Receptor-Negative Breast Cancer (ER-/PR-/HER2-), SD–Standard Deviation. *P-value<0.05 is significant.

### Definition of breast cancer subtypes

The receptor subtypes were categorized in the following manner: (a) HR+ (ER+ and/or PR+) and HER2-, (b) HR+ (ER+ and/or PR+) and HER2+, (c) HR- and HER2+, and (d) HR- and HER2- (TNBC) based on immunohistochemistry (IHC) analysis. The ER/PR, HER2, and Ki67 status were obtained from the patient’s pathology reports. HR+ was defined as >5% nuclear positive for ER and/or PR in tumor cells. HER2+ was defined as HER2 3+ by IHC and/or more than 2.2 HER2 genes counted for every copy of chromosome 17 (HER2/CEP17 ratio) by FISH analysis. Ki67 Low was defined as <= 20% nuclear positive, and Ki67 High was defined as > 20% nuclear positive. 

### IHC staining

We selected cases with available paraffin blocks and whose paraffin slides for tumor tissue contained more than 10% tumor cells. A total of 150 blocks were selected and IHC was performed as described in a previous study [17]. Briefly, antigen retrieval was performed by treating the tissue section with sodium citrate (10 mM, pH 6.0) at 95°C. The tissue was treated for 10 minutes for CD44 and CD24 staining, and 20 minutes for PTEN staining and subsequently cooled for 30 minutes at room temperature. CD44/CD24 expression was determined by double-IHC staining. CD44 (Ab-4, NeoMarkers; ready to use) was incubated for 60 minutes at room temperature (RT) and detected with Permanent Red (Vector Lab, CA). CD24 (Ab-2 (SN3b), NeoMarkers; 1:50 dilution) was incubated for 30 minutes at RT and detected using diaminobenzidene (DAB) (Vector Lab, CA). PTEN expression was determined by incubation of PTEN antibody (Clone 6H2.1, DAKO, 1:100 dilution) for 60 minutes at RT and then detected by using a Vectastain Universal *elite* ABC kit (PK-6200; Vector Laboratories, Burlingame, CA, USA) and visualized using DAB (Vector Lab, CA). FOXO1 and pAkt expression from same tissue sections were obtained from our previous studies [22,25]. Negative-control tests were conducted with the samples in the absence of primary antibody. Positive control paraffin slides with known negative or positive expression of CD24, CD44 or PTEN (IHC confirmed and antibody supplied by vendor) were tested alongside the unknown samples. Breast cancer cell lines, SKBR3, MCF7 were used for testing CD24 and PTEN antibodies, and MDA-MB231 was used for testing CD44 antibody before IHC staining of human samples. The proportion of CD44+/CD24- tumor cells was determined as the percentage of cells positive for Permanent Red staining but negative for DAB staining. The frequencies of other CD44/CD24 phenotypes were determined similarly. 

**Table 3:** CD44, CD24 Expression in Relation to Tumor Characteristics and Ki67, PTEN, pAkt, and FOXO1 Expression

**VARIABLE**			**CD44**		**CD24**		**CD44/CD24**		**CD24/CD44**	
			**Positive**	**Negative**	**Positive**	**Negative**	**Positive**	**Negative**	**Positive**	**Negative**
			N (%	N (%)	N (%)	N (%)	N (%)	N (%)	N (%)	N (%)
**ER status**										
	Positive		32(53.3)	44(66.7)	42(56.0)	34(66.7)	14(37.8)	62(69.7)	26(52.0)	50(65.8)
	Negative		28(46.7)	22(33.3)	33(44.0)	17(33.3)	23(62.2)	27(30.3)	24(48.0)	26(34.2)
			P-Value=0.147		P-Value=0.268		P-Value=0.001*		P-Value=0.139	
**PR status**										
	Positive		28(46.7)	36(54.5)	35(46.7)	29(56.9)	13(35.1)	51(57.3)	23(46.0)	41(53.9)
	Negative		32(53.3)	30(45.5)	40(53.3)	22(43.1)	24(64.9)	38(42.7)	27(54.0)	35(46.1)
			P-Value=0.476		P-Value=0.281		P-Value=0.031*		P-Value=0.467	
**HER2 status**									
	Positive		14(23.3)	20(30.3)	26 (34.7)	8(15.7)	7 (18.9)	27(30.3)	19(38.0)	15(19.7)
	Negative		46(76.7)	46(69.7)	49 (65.3)	43(84.3)	30(81.1)	62(69.7)	31(62.0)	61(80.3)
			P-Value=0.426		P-Value = 0.024*		P-Value=0.270		P-Value=0.039*	
**Ki67**										
	Low		30(51.7)	48(82.8)	43(63.2)	35(72.9)	16(44.4)	62(77.5)	31(68.9)	47(66.2)
	High		28(48.3)	10(17.2)	25(36.8)	13(27.1)	20(55.6)	18(22.5)	14(31.1)	24(33.8)
			P-Value=0.001*		P-Value=0.319		P-Value=0.001*		P-Value=0.840	
**Tumor size**										
	≤ 5 cm		41(70.7)	41(65.1)	44(62.9)	38(74.5)	26(72.2)	56(65.9)	28(60.9)	54(72.0)
	> 5 cm		17(29.3)	22(34.9)	26(37.1)	13(25.5)	10(27.8)	29(34.1)	18(39.1)	21(28.0)
			P-Value=0.562		P-Value=0.237		P-Value=0.531		P-Value=0.232	
**Lymph Node**										
	Negative		28(47.5)	27(42.9)	29 (40.8)	26(51.0)	18(50.0)	37(43.0)	19(40.4)	36(48.0)
	Positive		31(52.5)	36(57.1)	42 (59.2)	25(49.0)	18(50.0)	49(57.0)	28(59.6)	39(52.0)
			P-Value=0.716		P-Value = 0.276		P-Value=0.551		P-Value=0.458	
**PTEN**										
	Positive		30(52.6)	35(58.3)	35(48.6)	30(66.7)	12(35.3)	53(63.9)	22(44.9)	43(63.2)
	Negative		27(47.4)	25(41.7)	37(51.4)	15(33.3)	22(64.7)	30(36.1)	27(55.1)	25(36.8)
			P-Value=0.580		P-Value=0.06		P-Value=0.007*		P-Value=0.06	
**pAkt**										
	Negative		19(40.4)	29(51.8)	23(35.9)	25(64.1)	10(33.3)	38(52.1)	17(38.6)	31(52.5)
	Positive		28(59.6)	27(48.2)	41(64.1)	14(35.9)	20(66.7)	35(47.9)	27(61.4)	28(47.5)
			P-Value=0.322		P-Value=0.008*		P-Value=0.127		P-Value=0.170	
**FOXO1**										
	Positive		29(50.9)	26(43.3)	26(36.1)	29(64.4)	18(51.4)	37(45.1)	16(32.7)	39(57.4)
	Negative		28(49.1)	34(56.7)	46(63.9)	16(35.6)	17(48.6)	45(54.9)	33(67.3)	29(42.6)
			P-Value=0.461		P-Value=0.004*		P-Value=0.551		P-Value=0.009*	

Abbreviations: ER–Estrogen Receptor, PR– Progesterone Receptor. *P-value<0.05 is significant.

PTEN (Clone 6H2.1, DAKO) expression was determined by IHC staining for N=142 patients and described in a previous study [17]. Evaluation of PTEN was based exclusively on positive cytoplasm staining, and PTEN positive was defined as >5% positive in tumor tissue. FOXO1 and pAkt levels and methods for assessment in corresponding tissue sections are described in our previous studies [17,20,28]. Each tissue section was blocked with 5% normal horse serum for 30 minutes followed by overnight incubation at 4°C with antibodies specific for phosphor-Akt (Ser473) (pAkt) (#9271; Cell Signaling Technology, Inc., Danvers, MA, USA) and total Akt (#9272; Cell Signaling Technology, Inc.). Immunostaining was visualized with a streptavidin peroxidase reaction using the DAB (3,3'-diaminobenzidine) kit (SK-4100; Vector Laboratories). The nuclei were counterstained with hematoxylin before mounting. Negative-control tests were conducted with samples in the absence of primary antibody. Similarly, control paraffin slides with known negative or positive expression of pAkt (IHC confirmed and antibody supplied by vendor) were tested alongside the unknown samples.

### Evaluation of IHC staining

The CD44 staining was detected mainly in the cell membrane and some cytoplasm. The CD24 staining was detected mainly in cell membrane and cell nuclei. Light microscopy and digital computer software (DigiPro, Labomed, Inc., Culver City, CA, USA) were used to identify the protein staining intensity level and quantify the proportion of positive cells. The intensity of positive staining in tissue samples was scored using three pluses (+++) for high intensity staining, two pluses (++) for moderate staining, one plus (+) for low intensity and negative (no staining). The percentage of staining was categorized as: 0=negative, 1=<10% positive tumor cells, 2=11% to 50% positive cells, 3=51% to 80% positive cells; and 4=80% to 100% positive cells. Similar to our previous study the final quantification of IHC results for both variables (the intensity of the staining and the percentage cells with positive staining) was considered (score = intensity x positive) [22]. The proportion of CD44+/CD24- tumor cells was determined as the percentage of cells positive for Permanent Red staining but negative for DAB staining. The frequencies of CD44-/CD24+ cells and of CD44+/CD24+ cells were determined in a similar fashion. To control the reliability of the CD44 and CD24 double-staining, single-staining with CD44 and CD24 antibodies were also performed and used as reference for the scoring. 

**Table 4:** Cox-regression Analysis –Multivariate analysis

			MODEL-1*						**MODEL-2†**	
			**African-American**			**Hispanic**				
			**RR (95%CI)**	**P-VALUE**		**RR (95%CI)**	**P-VALUE**		**RR (95%CI)**	**P-VALUE**
**CD44+/CD24-**										
	Negative		1			1			1	
	Positive		3.6 (0.9-15.2)	0.082		1.6 (0.4-7.4)	0.523		2.9 (0.7-12.8)	0.149
**CD24+/CD44-**										
	Negative		1			1			1	
	Positive		1.9 (0.7-5.0)	0.193		0.8 (0.3-2.1)	0.613		0.4 (0.1-1.2)	0.091
**Ki67**										
	Low (<10%)		1			1			1	
	Moderate (10%-20%)		0.5 (0.1-2.1)	0.372		0.7 (0.1-6.0)	0.711		1.5 (0.4-6.0)	0.562
	High (>20%)		2.0 (0.7-5.3)	0.177		0.8 (0.2-2.9)	0.785		1.1 (0.4-3.0)	0.876
**PTEN**										
	Positive		1			1			1	
	Negative		2.5 (1.1-5.6)	**0.031**		0.8 (0.3-2.2)	0.641		0.5 (0.2-1.9)	0.325
**Receptor Subtype**										
	HR+/HER2-		1			1			1	
	HER2+		2.8 (1.2-6.5)	**0.019**		1.7 (0.7-4.1)	0.261		6.9 (1.8-26.5)	**0.005**
	TNBC		2.5 (1.0-6.2)	**0.041**		3.5 (1.3-9.5)	**0.014**		10.4 (5.2-24.1)	**<0.001**

* Model-1: Adjusted for Tumor size, Node involvement, Tumor stage, treatment, and Age for each marker or subtype

† Model-2: Included all markers and subtype into the model and adjusted for Tumor size, Node involvement, Tumor stage, treatment, Age, and Ethnicity.

Abbreviations: H -Hormonal Receptor (Estrogen /Progesterone Receptor), HER2 (human epidermal growth factor receptor 2),TNBC–Triple Receptor-Negative Breast Cancer (ER-/PR-/HER2-) , RR–Risk Ratio, CI–Confidence Interval.

Evaluation of PTEN positive was based exclusively on cytoplasm staining, although some nuclear uptake was also observed in a few cases. The percentage of cancer cells with cytoplasm staining was scored and cases with positive staining in more than 5% tumor cells were considered as positive. Each tissue was evaluated and scored by two clinical pathologists blinded to the origin of the tissue and only the tissue sections that had good tissue structure and clear staining were included for analysis. The final analyses included 126 cases assessed for CD44/CD24 and 142 cases assessed for PTEN. 

### Determination of IGF-I and IGFBP3 levels

Plasma IGF-I and serum IGFBP3 levels were measured by Radioimmunoassay (RIA) (Nicholls Institute Diagnostics) as described in our previous study [11]. Briefly, the acid-ethanol precipitation method was used to measure IGFBP-free IGF-I levels in plasma according to manufacturers’ instructions. For measuring IGFBP3 levels, serum samples were diluted 1:250 with assay buffer and RIA assay was performed according to manufacturers’ instructions.

### Statistical analysis

All of the analyses were performed with a statistical package, SPSS (11.5, SPSS Inc., Chicago, IL, USA). Kaplan-Meier survival-curves with log-rank testing were used to assess the Disease Free Survival (DFS). The relative risk (RR) of shorter disease-free-survival was determined by Cox-regression with univariate and multivariate analysis. ANOVA and chi-square tests were used to compare the quantitative or categorical variables respectively. A p-value < 0.05 was considered statistically significant.

## Results

### Clinical/Demographic characteristics

Among the study participants, 52% were African-American women and 48% were Hispanic/Latina. The mean age at the time of diagnosis was 52 years for African-American and 49 years for Latina women (Table 1). The distribution of receptor subtypes was as follows: 49% HR+/HER-, ~9% HR+/HER2+, ~10% HR-/HER2+ and ~32% TNBC. Detailed clinical and demographic information on the different types of tumors are summarized in Table 1. The most prevalent subtype was HR+/HER2- in both African-Americans (49.4%) and Latinas (48.7%) followed by TNBC at 35.0% for African-Americans and 28.3% for Latinas. The prevalence of HR-/HER+ subtype was more frequent in Latina than African-American women (13.8% vs. 6.6%). As shown in Table 1, tumor subtypes differed significantly by age (p=0.011), tumor size (p=0.022), and histological grade (p<0.001). Latinas diagnosed at age<50 also had higher prevalence of HR-/HER2+ subtype. TNBCs were more likely to be among younger women, be poorly differentiated, and have higher Ki67 index. 

**Table 5:** Prevalence of the Triple-Negative Receptor Breast Cancer Subtype by Study and Ethnicity

**STUDY (REFERENCE)**		**STUDY-DATASET**		**SAMPLE SIZE**		**ETHNICITY**				**P-VALUE**
				**Ethnicity = (N)**		**NHW**	**AA**	**Hisp**		
Millikan, 2008 [43]		Carolina Breast Cancer Study (CBCS)		NHW=843		21(%)	35(%)	-		n/a
				AA=581						
Stead, 2009 [7]		Boston University Medical Center		NHW=148		13(%)	30(%)	12(%)		0.0002*
				AA=177						
				Hisp=43						
Lund, 2010 [8]		Atlanta Surveillance, Epidemiology,& End Results (SEER) Registry		NHW=96		10(%)	23(%)	-		<0.001*
				AA=814						
Komenaka, 2010 [44]		Wishard Memorial Hospital, Indianapolis, Indiana		NHW=315		19(%)	32(%)	-		0.05*
				AA=315						
Parise, 2010 [45]		California Cancer Registry (CCR)		NHW=48863		12(%)	28(%)	18(%)		n/a
				AA=3743						
				Hisp=9588						
Stark, 2011[46]		Henry Ford Hospital, Detroit, Michigan		NHW=1008		16(%)	26(%)	-		n/a
				AA=581						
Hines, 2010 [3]		4-Corners Breast Cancer Study, Colorado		NHW=119		15(%)	-	17(%)		n.s.
				Hisp=69						
Lara-Medina, 2011[47]		National Cancer Institute		Hisp=2065		-	-	23(%)		--
Vadgama, 2013		Martin Luther King Ambulatory Care Center (MACC)		AA=166		-	35(%)	28(%)		n.s
				Hisp=152						

Abbreviations: NHW - Non-Hispanic-White/Caucasian, AA- African-American, Hisp-Hispanic/Latina, n.s. – Not Significant (P>0.05), n/a-information is not available *P-value<0.05 is significant.

### Expression of CD44, CD24 and other Biomarkers

**Figure 1:**
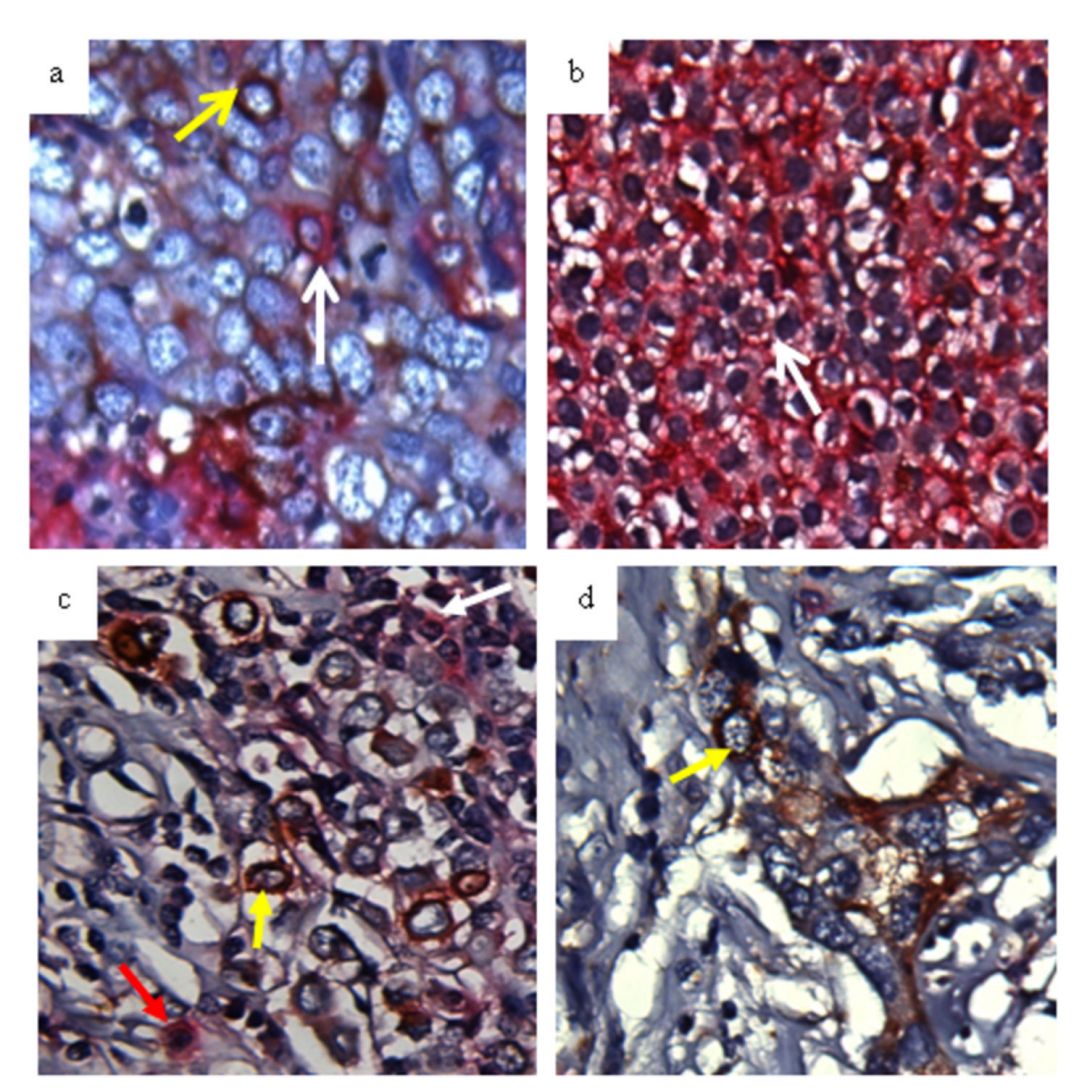
Expression pattern of CD44 and CD24 in breast cancer tissue. Breast tissues were double-stained with antibodies specific for CD44 and CD24. CD44 was detected with Permanent Red and CD24 was detected using diaminobenzidene (DAB brown). The white arrow indicates CD44+ cells (a and b), the yellow arrow indicates CD24+ cells (a, c, d), and the red arrow indicates CD44+/CD24+ cells (c).

Figure 1 demonstrates an example of tumor cells positively stained for CD44 (Permanent Red, Figure 1b) and CD24 (DAB brown, Figure 1d). In tissue sections, some tumor cells were CD44+ and others were CD24+ (Figure 1a). We also observed that some tumor cells were both CD44+ and CD24+ (Figure 1c). The frequency of tumor cells expressing CD44 and CD24 is summarized in Table 2. The HR+/HER2- tumors were more likely to be CD44- compared to HER2-positive (HR+/HER2+ and HR-/HER2+) and TNBC tumors. The prevalence of CD44+/CD24- phenotype was significantly higher in TNBC than that in other subtypes. There was no difference in the prevalence of CD44 and CD24 phenotypes between African-Americans and Latinas. Table 2 also shows that PTEN loss is more frequent in TNBC, and reduced FOXO1 is more related to the HR-/HER2+ subtype. An early study from our laboratory has demonstrated that high plasma IGF-I (>120 ng/ml) was associated with decreased survival rate in younger African-American and Latina women [11]. In this study, premenopausal women had higher IGF-I levels (p= 0.0318) than postmenopausal women (Table 2). TNBC group had increased IGF-I levels compared to the other subtypes, however, the difference did not reach to statistical significance. The IGFBP3 levels were similar among the participants. 

**Table 6:** Association of CD44+/CD24- Phenotype and Disease Outcome by Study and Multivariate Analysis.

**STUDY (REFERENCE)**		**SAMPLE**			**CD44+/CD24-**		**REDUCED DFS†**		**MULTIVARIATE ANALYSIS††**	
		**(N)**	**Ethnicity**		**%**		**P-VALUE**		**HR††† (95% CI)**	**P-VALUE**
Lin, 2012 [34]		147	Asian		70		0.001*		2.2 (1.3-3.7)	0.002*
Perrone, 2012 [36]		56	Caucasian		91		<0.05 *		6.0 (1.8-19.9)	0.003*
Xu, 2012 [37]		1086	Asian		31		n.s		1.2 (0.8-1.7)	0.32
Giatromanolaki, 2010 [27]		139	Caucasian		29		n.s.		1.3	0.18
Lee, 2011 [33]		92	Asian		40		0.043*		-	-
Honeth, 2008 [26]		240	Caucasian		31		n/a		-	-
Mylona, 2008 [35]		155	Caucasian		59		0.074		-	-
Abraham, 2005 [24]		136	Caucasian		22		n.s.		-	-
Kim, 2011 [38]		643	Asian		21		0.003 *		0.7 (0.5-0.9)	0.02*
Vadgama, 2013		126	African-American, Hispanic		29		0.05 *		1.0 (0.5-2.1)	0.9

Abbreviations: DFS-Disease Free Survival, HR-Hazard Ratio, CI–Confidence Interval, n.s–Not Significant (P>0.05). n/a-information is not available, AA-African-American, Hisp-Hispanic/Latina.

* P-value<0.05 is significant.

† Kaplan-Meier Survival analysis with Log-rank test.

†† Adjusted for Tumor Clinicopathology and Age.

††† Hazard Ratio for Reduced DFS.

### CD44, CD24 expression in relation to tumor characteristics and PTEN, pAkt, FOXO1 expression

Table 3 demonstrates that the individual ER, PR or HER2 status is not associated with CD44 or CD24 expression, however, the CD44+/CD24- phenotype is associated with ER- and PR- tumors (p=0.001 and p=0.031, respectively). The CD44+ and the CD44+/CD24- phenotypes are also associated with high Ki67 expression (p=0.001). PTEN loss is also associated with the CD44+/CD24- phenotype (p=0.007). The expression of CD24+ and the CD24+/CD44- phenotype was significantly higher in HER2+ tumors (p=0.024 and p=0.039, respectively) and tumors with low FOXO1 expression (p=0.004). High pAkt expression was associated (p=0.008) only with the CD24+ phenotype (Table 3).

### Disease-Free-Survival (DFS)

**Figure 2:**
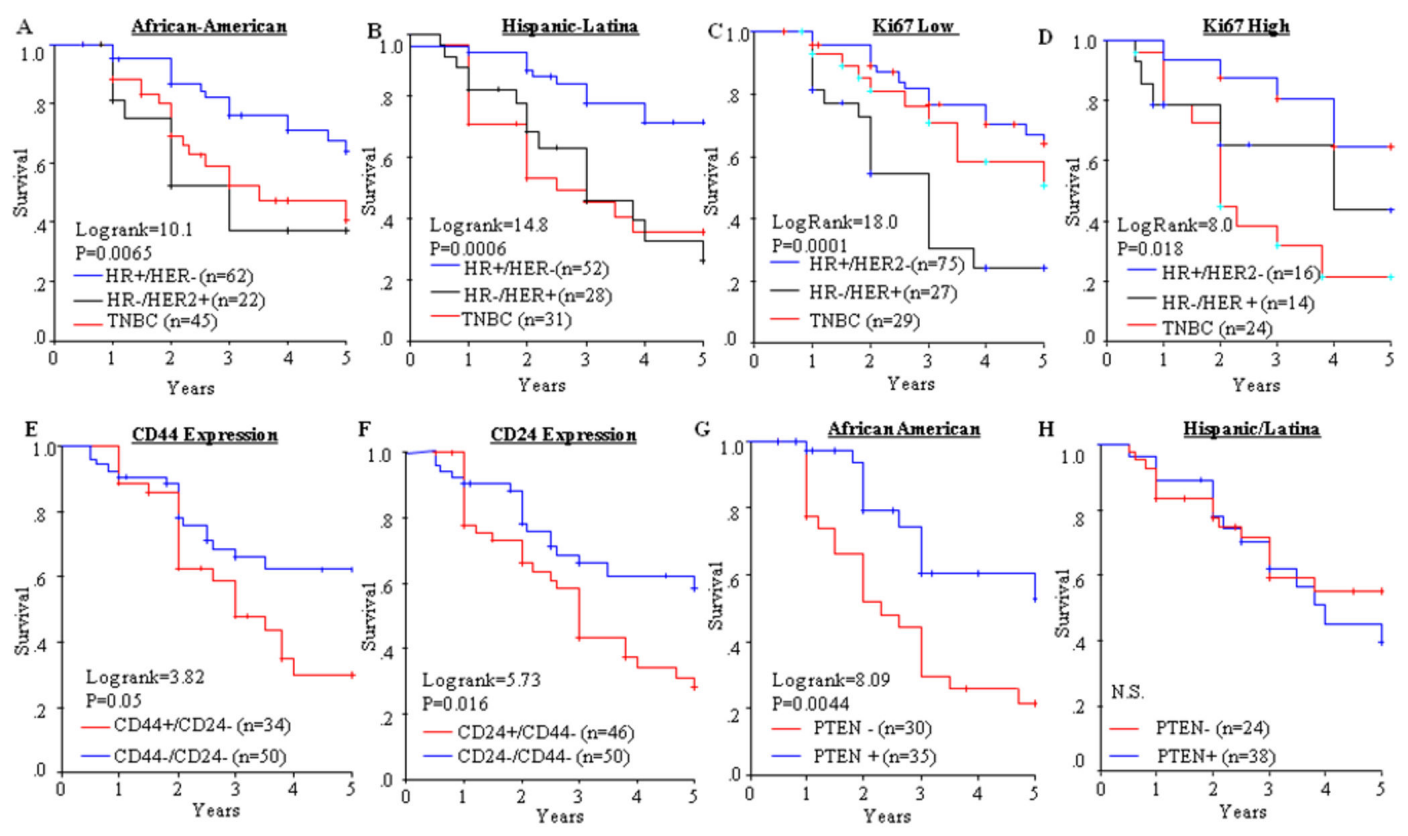
Disease Free Survival (DFS) and breast cancer subtypes. Kaplan-Meier was used to compare the 5-year DFS among cancer subtypes in: (A) African-Americans; (B) Hispanic/Latinas. (C) low Ki67; (D) high Ki67; (E) CD44+/CD24- vs. CD44-/CD24-; (F) CD24+/CD44- vs. CD24-/CD44-; (G) PTEN in African-Americans; and (H) PTEN in Hispanic/ Latinas. Log-rank test was used to determine the significance between the curves.

African-American and Latina women with TNBC and HER2+ tumors had significantly shorter DFS compared with the HR+ tumors (Figure 2A/2B). Upon stratification by Ki67 (Figure 2C/2D), patients with HER2+ disease showed lower DFS in the Ki67 low group and patients with TNBC had lower DFS in the Ki67 high group. Overall, patients with the CD44+/CD24- or CD24+/CD44- phenotypes experienced significantly reduced DFS (Figure 2E/2F). Interestingly, loss of PTEN reduced DFS significantly in African-Americans, but not in Latinas (Figure 2G/2H). Since the DFS may be influenced by treatment and other tumor characteristics, Cox regression with multivariate analysis was performed. 

More than 80% of patients in this cohort had gone through chemotherapy for treatment of the primary tumor. However, African-American women were less likely to have chemotherapy compared to Latinas (78% vs. 91%) overall. Around 50% of African-American (51%) and Latina patients (55%) received CAF (Cyclophosphamide, Adriamycin, and Fluorouracil) or CMF (Cyclophosphamide, Methotrexate, and Fluorouracil) based chemotherapy. Around 27% of African-American and 35% of Latina women had CAF/CMF plus Taxol. Overall 73% patients with HR+ tumor had hormone-therapy (African American=75% and Latina=71%). The majority of women received Tamoxifen and few received Aromatase inhibitors (^≈^3%). In addition, most of the patients with HER2 positive tumor did not receive trastuzumab since many of them were diagnosed with breast cancer before 2006. The RR for each biomarker and receptor subtype were assessed and adjusted for treatment type and tumor characteristics. 

Data from Model-1 analysis showed that loss of PTEN impacted DFS significantly in African-Americans, but not in Latinas. The TNBC phenotype was associated with higher RR in both African-American and Latina women, but HER2+ status increased RR in African-Americans only. Expression of CD44/CD24 phenotypes and Ki67 were not significantly related to RR. Model-2 in Table 4 presents analysis including all biomarkers and receptor subtypes adjusted for treatment, tumor size, node status, stage, age and ethnicity (Model-2 in Table 4). In Model-2 only the tumor receptor subtype classifications were significantly associated with DFS (Table 4). 

## Discussion

 This study has provided additional information reporting frequencies of receptor subtypes in African-American and Hispanic women in South Los Angeles. Results from a literature review are presented in Table 5 and confirm that pooled overall prevalence of TNBC was significantly higher in African-American (~28%, range from 23% to 35%) and Hispanics (~19%, range 12% to 23%) compared with Caucasian women (~12%, range from 12% to 21%). The prevalence of TNBC in African-Americans in this study was similar to the data reported by Millikan et al (35%), but higher than in the overall assessment (Table 5). The prevalence of TNBC in Hispanics in the present study was higher than previously reported by other studies, as well as in Caucasians (Table 5). The younger age of breast cancer onset in this study cohort could partially explain the high prevalence of TNBC observed in our study compared with other reports. An inverse association between TNBC and age at diagnosis has been reported in multiple studies [2,6]. This may be a significant factor affecting cancer health disparities observed in underrepresented patient population, particularly in South Los Angeles.

Our study is among the first to report clinical association between TNBC and PTEN loss (Table 2) in underrepresented women [29]. PTEN is one of the key regulators of the PI3K/Akt pathway activation, acting as a tumor suppressor inhibiting Akt phosphorylation. The loss of PTEN has been associated with poor response to treatment and disease outcome and substantial increase in Akt signaling [30]. Therefore, PTEN loss in TNBC may also help explain the reduced survival in these patients. Interestingly, PTEN loss was significantly associated with reduced DFS in African-American women after adjusting for treatment and other tumor features, but not significant among the Latina women in our cohort. Furthermore, the high levels of IGF-I among premenopausal women, particularly within the TNBC cohort, may possibly compound over-activation of PI3K/Akt by binding to IGF-IR and initiating additional Akt pathway activation. Therapeutically, there are several PI3K/Akt specific inhibitors in the clinical trials phase, with several such as BKM120 and MK-2206 showing significant promise for breast cancer treatment [31,32]. The findings from our study identify a cohort of African-American women with TNBC, high prevalence of PTEN loss, and previous indication of high pAkt activation [17] may benefit from such treatment. 

To our knowledge, our study is one of the first to report patient survival in relation to the CD44/CD24 phenotypes among African-American and Hispanic/Latina women [24,26,27,33-38]. Table 6 shows an ethnic-specific composite of studies which had a sample size>50 and CD44/CD24 determined by double staining. The 29% prevalence of the CD44+/CD24- phenotype in the present study is similar to ~33% reported in the overall pooled proportion (Table 6). Significantly shorter DFS in women with the CD44+/CD24- phenotype was reported in 4/9 studies, and is consistent with findings in this study (P=0.05). Notably, our study identified that the CD44+/CD24- phenotypes were significantly associated with TNBC (Table 2 and Table 3), which complement previous reports among other ethnic groups [26,27,34,36]. There is a plethora of data in the literature building a strong association of CD44+/CD24- with “cancer stem-cell (CSC) like” or “mesenchymal” phenotype [22,39-41]. Future studies should aim to identify the mechanistic value of pathways associated with these biomarkers in order to potentially develop targeted CSC therapies. 

Our data also showed for the first time that the CD24+ or CD24+/CD44- phenotypes were more associated with the HER2-positive subtype. HER2-positive tumors have been correlated with decreased Ki67, increased pAkt, and decreased FOXO1 [17,20]. Reduced FOXO1 has also been shown to contribute to trastuzumab resistance [28]. Interestingly, the HR-/HER2+ patients with low Ki67 expression also had lower DFS (Figure 1). Possible mechanisms accounting for this observation may be that anti-HER2 therapy (trastuzumab) is often combined with chemotherapeutic agents targeting cell cycle processes and highly proliferative cells to create a “synergistic” response [42]. Tumor cells with low proliferation (low Ki67) may not respond to chemotherapies directed towards mitotic inhibition, and subsequently, result in more rapid cancer progression and lower DFS. The specific role of the CD24+ phenotype in relation to these processes must be investigated further. 

Lastly, this study identified that patients with TNBC tend to have higher plasma IGF-I levels (Table 2). Studies have shown that BRCA1 germline mutation was more frequent in TNBC or basal-like subtype tumors and also associated with the CD44+/CD24- phenotype [34]. We have not yet investigated the BRCA1 status in our TNBC patients, and therefore are unable to confirm a relationship between BRCA1 and the CD44+/CD24- phenotype. Our previous study had shown that early onset African-American and Hispanic/Latina breast cancer patients did not express increased frequency of BRCA1 mutations, in particular the BRCA1 185AG deletion [43]. Additional studies will be conducted investigating the potential overlap of the IGF axis, BRCA1, and CSC phenotypes, including with additional markers such as ALDH1, within the existing panel to better refine associations among African-American and Hispanic women.

In conclusion, this study reported the prevalence of tumor subtypes among African-American and Hispanic women and identified that the TNBC and HR-/HER2+ were significantly high in these populations and associated with poor outcome. Notably, this study has reported that the loss of PTEN is highly prevalent in TNBC, and is associated with poor DFS among African-American women after adjustment for treatment and other tumor pathological markers. Multiple agents such as BKM120 and MK-2206 for PI3K/Akt inhibition are currently at the clinical trials level; however, they are being explored largely within the context of HR+ or HER2+ disease. The correlation of PTEN loss suggests that these agents could possibly benefit TNBC patients, especially since there are limited therapeutic options available for this breast cancer subtype. The high prevalence of TNBC among underrepresented populations such as African-American women is a strong contributor to breast cancer health disparities, hence improving targeted therapies could make a significant impact in this area. Further elucidation of the role of these protein profiles, particularly the role of the CSC phenotype, will improve our understanding of the development and progression of breast cancer, and ultimately aid in improving outcomes among populations particularly affected by cancer health disparities. 
